# An Overview of the Role of Long Non-Coding RNAs in Human Choriocarcinoma

**DOI:** 10.3390/ijms22126506

**Published:** 2021-06-17

**Authors:** Riccardo Di Fiore, Sherif Suleiman, Ana Felix, Sharon A. O’Toole, John J. O’Leary, Mark P. Ward, James Beirne, Maja Sabol, Petar Ozretić, Angel Yordanov, Mariela Vasileva-Slaveva, Stoyan Kostov, Margarita Nikolova, Ian Said-Huntingford, Duncan Ayers, Bridget Ellul, Francesca Pentimalli, Antonio Giordano, Jean Calleja-Agius

**Affiliations:** 1 Department of Anatomy, Faculty of Medicine and Surgery, University of Malta, MSD 2080 Msida, Malta; sherif.s.suleiman@um.edu.mt; 2 Center for Biotechnology, Sbarro Institute for Cancer Research and Molecular Medicine, College of Science and Technology, Temple University, Philadelphia, PA 19122, USA; president@shro.org; 3 Department of Pathology, Campo dos Mártires da Pátria, Instituto Portugues de Oncologia de Lisboa, NOVA Medical School, UNL, 130, 1169-056 Lisboa, Portugal; ana.felix@nms.unl.pt; 4 Departments of Obstetrics and Gynaecology and Histopathology, Trinity St James’s Cancer Institute, Trinity College Dublin, 8 Dublin, Ireland; shotoole@tcd.ie; 5 Department of Histopathology, Trinity College Dublin, Trinity St James’s Cancer Institute, 8 Dublin, Ireland; olearyjj@tcd.ie (J.J.O.); wardm6@tcd.ie (M.P.W.); 6 Department of Gynaecological Oncology, Trinity St James Cancer Institute, St James Hospital, 8 Dublin, Ireland; JBeirne@stjames.ie; 7 Laboratory for Hereditary Cancer, Division of Molecular Medicine, Ruđer Bošković Institute, 10000 Zagreb, Croatia; maja.sabol@irb.hr (M.S.); pozretic@irb.hr (P.O.); 8 Department of Gynecologic Oncology, Medical University Pleven, 5800 Pleven, Bulgaria; angel.jordanov@gmail.com; 9 Department of Surgery, Alexandrovska University Hospital, 1431 Sofia, Bulgaria; sscvasileva@gmail.com; 10 Department of Gynecology, Medical University Varna “Prof. Dr. Paraskev Stoyanov”, 9002 Varna, Bulgaria; drstoqn.kostov@gmail.com; 11 Saint Marina University Hospital—Pleven, Medical University Pleven, 5800 Pleven, Bulgaria; mnikol@abv.bg; 12 Department of Histopathology, Mater Dei Hospital, Birkirkara Bypass, MSD 2090 Msida, Malta; ian.said-huntingford@gov.mt; 13 Centre for Molecular Medicine & Biobanking, University of Malta, MSD 2080 Msida, Malta; Duncan.Ayers@um.edu.mt (D.A.); bridget.ellul@um.edu.mt (B.E.); 14 Faculty of Biology, Medicine and Human Sciences, The University of Manchester, Manchester M1 7DN, UK; 15 Cell Biology and Biotherapy Unit, Istituto Nazionale Tumori-IRCCS-Fondazione G. Pascale, 80131 Napoli, Italy; f.pentimalli@istitutotumori.na.it; 16 Department of Medical Biotechnologies, University of Siena, 53100 Siena, Italy

**Keywords:** choriocarcinoma, rare cancer, long non-coding RNA, oncogenes or tumor suppressor genes, lncRNA-based therapy

## Abstract

Choriocarcinoma (CC), a subtype of trophoblastic disease, is a rare and highly aggressive neoplasm. There are two main CC subtypes: gestational and non-gestational, (so called when it develops as a component of a germ cell tumor or is related to a somatic mutation of a poorly differentiated carcinoma), each with very diverse biological activity. A therapeutic approach is highly effective in patients with early-stage CC. The advanced stage of the disease also has a good prognosis with around 95% of patients cured following chemotherapy. However, advancements in diagnosis and treatment are always needed to improve outcomes for patients with CC. Long non-coding (lnc) RNAs are non-coding transcripts that are longer than 200 nucleotides. LncRNAs can act as oncogenes or tumor suppressor genes. Deregulation of their expression has a key role in tumor development, angiogenesis, differentiation, migration, apoptosis, and proliferation. Furthermore, detection of cancer-associated lncRNAs in body fluids, such as blood, saliva, and urine of cancer patients, is emerging as a novel method for cancer diagnosis. Although there is evidence for the potential role of lncRNAs in a number of cancers of the female genital tract, their role in CC is poorly understood. This review summarizes the current knowledge of lncRNAs in gestational CC and how this may be applied to future therapeutic strategies in the treatment of this rare cancer.

## 1. Introduction

**Figure 1:**
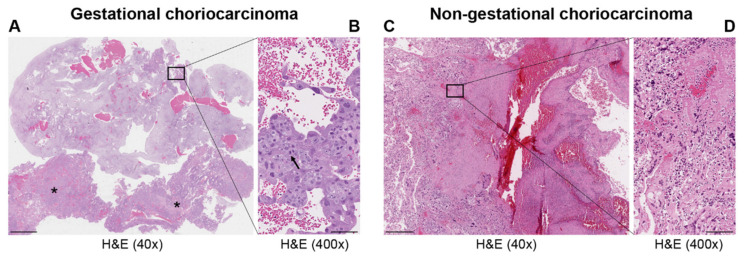
Representative images of hematoxylin and eosin (H&E) staining in gestational (**A**,**B**) and non-gestational (**C**,**D**) choriocarcinoma. Uterine curettage (**A**,**B**); (**A**) low power (40×; scale bar = 500 μm) with abundant decidualized endometrium and blood and two large clusters of choriocarcinoma (*); (**B**) high power (400×; scale bar = 50 μm) shows aggregates of large trophoblasts with marked atypia and prominent mitotic figures (arrow) covered by atypical syncytiotrophoblasts. Invasive choriocarcinoma in the ovary (**C**,**D**). (**C**) Component of a germ cell tumor of the ovary with large and atypical trophloblastic cells with hemorrhage and necrosis (low power 40×; scale bar = 500 μm) associated with dysgerminoma (not present in the picture). (**D**) Markedly atypical synciotrophoblasts and cytotrophoblasts (high power 400×; scale bar = 50 μm).

Choriocarcinoma (CC), a subtype of trophoblastic disease [1], is a rare and highly aggressive tumor with varied global incidence [2]. CC is histologically characterized by an admixture of: syncytiotrophoblasts and mononucleated cytotrophoblastic cells. Two significant CC subtypes, gestational and non-gestational (so called when it develops as a component of a germ cell tumor or is related to a somatic mutation of a poorly differentiated carcinoma) (Figure 1), have diverse underlying biologies and clinical outcomes (Table 1) [3]. The former can arise in the uterus and rarely in extrauterine sites following a hydatidiform mole (50%), a miscarriage or induced abortion (25%), a normal pregnancy (22.5%), or an ectopic pregnancy (2.5%) [2]. Non-gestational CCs have been shown to arise from pluripotent germ cells in both male and female gonads, most commonly in the ovary and retroperitoneum [3,4,5], or pelvis [6]. Pure CC is exceedingly rare, but focal areas of CC are seen in approximately 12% of embryonal and teratocarcinomas [7]. Foci of CC may be present in gestational mixed trophoblastic tumors and in non-gestational mixed germ cell tumors. Rare cases of extrauterine carcinoma with trophoblastic cells represent differentiation of pluripotent cells into malignant somatic cells [3].

As non-gestational CC has no specific genetic features but has a similar appearance to gestational CC without any of its special characteristics, hence, we will only focus on gestational CC, which has unique biological characteristics.

To date, little is known regarding the underlying pathophysiology and oncogenesis in this malignancy. Gestational CC is likely to arise as a result of aberrations of methylation during development, rather than from DNA mutations, supporting the hypothesis that it arises from normally transient early trophoblast cells [10]. At a molecular level, gestational CC is also characterized by an overexpression of *TP53*, *MDM2*, and epidermal growth factor receptor (*EGFR*), and downregulation of a number of genes, including *NECC1*, *DOC-2/hDab2*, *KRAS*, *CDH1*, *CDKN2A*, *HIC-1,* and *TIMP3* [8]. Other oncoproteins (BCL-2, c-FMS, c-ERB-2, and c-MYC) exhibiting synergistic upregulation have also been implicated in the pathogenesis of CC [8]. Moreover, elevated levels of human leucocyte antigen-G (HLA-G) in CC may inactivate the local immune system, thus altering the tumor microenvironment as well as promoting proliferative and metastatic capability of the tumor [8]. Jung et al. detected driver mutations in gestational CC, most of which were chromatin remodeling gene mutations (*ARID1A*, *SMARCD1*, and *EP300*) [9]. A heterozygous germline mutation was also found in the *NLRP7* gene, which has been studied extensively in relation to gestational trophoblastic disease, with mutations of this gene having been reported in 50% of complete hydatidiform moles with high risk of evolving to CC [11]. Moreover, *NLRP7* has been shown to be involved in placental development by demonstrating its effects on trophoblast proliferation, differentiation, migration, invasion, and apoptosis [12].

A therapeutic approach is highly effective in patients with early-stage CC, and the advanced stage of the disease also has a good prognosis, with around 95% of patients being cured with chemotherapy. However, advances in diagnosis and treatment are always needed to improve outcomes for patients with CC [13].

Although the vast majority of the human genome is transcribed, only 2% of all the transcribed genes are protein-coding genes. There has been speculation over the role of non-coding RNA (ncRNA), ranging from junk transcripts to master epigenetic regulators. Long non-coding RNAs (lncRNAs) are a particular class of ncRNAs that have been increasingly recognized as having a fundamental regulatory role in both health and disease [14,15,16,17]. LncRNA transcripts are involved in transcriptional regulation, subcellular localization, and epigenetic remodeling. Dysregulated lncRNAs have been associated with cancer through either up- or downregulation of specific lncRNAs occurring relative to the adjacent normal tissue [18]. Therefore, these lncRNAs behave like oncogenes or tumor suppressor genes. For instance, overexpression of the *HOTAIR* lncRNA correlates with cancers that are more aggressive [19,20,21,22,23,24,25], whereas *MEG3* lncRNA may act as a tumor suppressor and its downregulation has been associated with the development of a variety of human cancer involving the liver, breast, uterus, and ovary [26]. LncRNAs can also contribute to the cellular fate programs in cancer stem cells, which are involved in tumorigenesis and therapy resistance [27]. OCT4 is a stemness-related transcription factor, which regulates the expression of both the lncRNAs *NEAT1* and *MALAT1* to promote the progress of lung cancer [28]. Moreover, *NEAT1* leads to stem-like phenotypes in NSCLC, TNBC, and GBM cells [29,30,31]. The lncRNA *B4GALT1-AS1* recruits the yes-associated protein (YAP) to the nucleus, a potent oncogene related to several oncogenic programs, including stemness. This enhances its transcriptional activity, thus further promoting stemness in colon cancer cells [32,33]. Another example has been shown in glioma, where the lncRNA *SNHG20* enhances stemness by activating the PI3K/Akt/mTOR signaling pathway [34].

Detection of lncRNAs associated with cancer in body fluids (i.e., blood, saliva, urine, etc.) is a valuable method not only for more effective cancer diagnosis, but possibly also for earlier detection of cancers and for use as therapeutic targets. Furthermore, using body fluids to detect circulating lncRNAs is much less invasive when compared to collecting biopsies [35].

Recently, Hosseini et al. published a comprehensive overview on the potential roles of lncRNAs in several cancers of the female reproductive system [36]. However, their role in CC is relatively poorly established. In this review, we summarize the current knowledge of lncRNA function and how this may be applied to future therapeutic strategies, specifically in the management of gestational CC.

## 2. Biological Characteristics of LncRNAs and Their Molecular Functions

LncRNAs are regulatory transcripts that are over 200 nucleotides long. These are mainly transcribed by RNA polymerase II, typically by a 5′7-methylguanosine cap and a 3′ poly (A) tail similar to messenger RNAs [37]. Non-coding transcripts (>200 nucleotides) which are generated from introns, exons, intergenic regions, telomeres, enhancers, or promoters are considered as different classes of lncRNAs (Table 2) [38,39,40,41,42]. Characteristically, lncRNAs are able to shuttle to numerous subcellular locations. There are very different levels of accumulation in the nucleus of certain lncRNAs when compared to cytoplasmic levels, while there is equal distribution for other lncRNAs [43]. LncRNAs are cell type dependent and the precise number of lncRNAs generated from the human genome is estimated to be very high (tens of thousands and up to more than one hundred thousand transcripts) [44,45]. Although most of these transcripts have not been studied, there is evidence that lncRNAs are able to regulate gene expression networks via the control of nuclear architecture and transcription in the nucleus, as well as the modulation of mRNA stability, together with translation and post-translational modifications in the cytoplasm [46]. This occurs mainly using four functional modes of action (Table 3) [47,48,49,50,51,52,53,54].

At the molecular level, the production of lncRNAs can act as a “signal” in response to a significant biological event, such as DNA damage. LncRNA transcripts can also regulate downstream functions that are related to other functional models. LncRNAs may also behave as a “decoy” through interaction with proteins and/or other RNA molecules, directly interfering with protein/genomic DNA, protein–protein, or RNA–RNA interactions. The decoy function of lncRNAs may sequester miRNA, preventing miRNA-mediated silencing of target mRNA. This is known as the miRNA sponging effect by lncRNAs. In addition, lncRNAs can serve as “guides” facilitating the recruitment of protein complexes to genomic loci. The lncRNA “scaffolds” act as the nucleation point, leading to the formation of protein complexes, or else mediate interaction between the different protein complexes [55]. Additionally, lncRNAs are involved in cancer chemoresistance [56,57].

## 3. Dysregulated lncRNAs in Choriocarcinoma

LncRNAs are crucial molecules in different biological processes and play an integral part in gene expression and its regulation. While there is still a lack of understanding of the function of lncRNAs, there is evidence that they are involved in the initiation and progression of a number of cancers [58,59]. LncRNAs, by acting as tumor suppressor genes or oncogenes, play a critical physiological role in apoptosis, invasion, metastasis, and cell proliferation in several cancers. LncRNAs are also involved in the pathogenesis of cancers of the female reproductive system (ovarian, uterine, vaginal, cervical, and vulvar cancers) [36]. However, to date, only a few studies address the role of lncRNA in CC. Novel mechanistic insights into how gene expression is specifically regulated by lncRNAs, contributing to CC formation, are outlined below. Table 4 summarizes the functions of the main lncRNAs implicated in CC.

### 3.1. MALAT1

Metastasis-associated lung adenocarcinoma transcript 1 (*MALAT1*), or, as it is also known, non-coding nuclear-enriched abundant transcript 2 (*NEAT2*), is a lncRNA which is 8000 nt long and is located on chr11q13.1 [68]. It has been shown to be involved in several physiological regulatory processes, including nuclear organization, epigenetic modification of gene expression, and alternative splicing via the modulation of phosphorylation of the SR splicing factor. It is also directly related to various pathological processes in non-communicable diseases, such as diabetes, and even in cancer [69]. The upregulation of *MALAT1* has been observed in different cancer types, including endometrial and cervical cancer, where there is an association with increased tumorigenesis and reduced survival [70,71,72]. In gastric cancer, it has been demonstrated that *MALAT1* can enhance *SOX2* mRNA stability, thus promoting stemness in cancer cells [27]. In CC, *MALAT1* might also promote tumor growth via miR-218-mediated *FBXW8* regulation [60]. This suggests that it could be therapeutically targeted in human CC.

### 3.2. H19

*H19* is a paternally imprinted gene, which encodes a 2300 nt long lncRNA. It is located on chromosome 11p15.5 and was identified from the transcription of the H19/insulin-like factor 2 gene cluster [73,74]. It has been shown that *H19* is upregulated only during the early stages of embryogenesis, and is downregulated after birth [75]. Hao et al. suggested that *H19* may act as a tumor suppressor gene [76], however, other studies reported an increased expression in several cancers [77,78]. *H19* regulates the expression of some cancer-related proteins, such as the ubiquitin ligase E3 family, calneuron 1 and retinoblastoma tumor suppressor (RB1) [79], and alpha-4, beta-3, and beta-5 integrins [80]. The dysregulation of *H19* in gynecological cancers (ovarian, endometrial, and cervical cancer) is associated with several molecular pathways that are normally disrupted in cancer [36]. In CC, there is an abnormal expression of *H19* where it plays an important role in tumor development [62]. Yu et al. investigated the role of *H19* in CC cells which are resistant to drugs and demonstrated that *H19*, through the regulation of the PI3K/AKT/mTOR pathway, results in drug resistance together with increased proliferative, migratory, and invasive ability of CC cells [61]. This suggests *H19* may be a potential therapeutic target for the treatment of drug-resistant CC.

### 3.3. MEG3

Maternally expressed gene 3 (*MEG3*) is a lncRNA which is 1600 nt long. It is located at the locus of *DLK1-MEG3* on human chromosome 14q32.3 [81]. *MEG3* is ubiquitously expressed in normal tissue and loss of its expression has been reported in various cancers, suggesting *MEG3* can behave as a tumor suppressor. When compared to normal tissue, there is a decreased expression of *MEG3* in ovarian cancer. *MEG3* overexpression causes anti-proliferative and cytotoxic effects in the OVCAR3 ovarian cancer cell line [26,81]. Likewise, *MEG3* is also downregulated in cervical cancer when compared to the adjacent normal tissues, and there is a negative correlation with tumor size, FIGO stage, lymphatic metastases, infection with human papilloma virus (HPV), and miR-21 expression [82]. Furthermore, cervical cancer cells undergo increased apoptosis and growth suppression after *MEG3* upregulation, confirming its role in tumor suppression in this type of cancer. Zhang et al. reported that CC progression in the human JEG-3 cell line was directly linked to a reduction in *MEG3* levels [63].

### 3.4. PCA3

Prostate cancer antigen 3 (*PCA3*) is a lncRNA that is located on chromosome 9q21-22 in the antisense direction in intron 6 of the prune homolog 2 gene (*PRUNE2* or *BMCC1*) [83]. *PCA3* is significantly upregulated in prostate cancer [84,85]. Increased expression of *PCA3* increases the proliferative, invasive, and migratory ability of prostate cancer cells and it is currently used as a diagnostic tool in managing prostate cancer [86,87]. Quek et al. also found increased expression of *PCA3* in epithelial ovarian cancer [88]. The expression of *PCA3* is upregulated in CC cells [64]. Furthermore, through sponging miR-106b, *PCA3* promotes the expression of MMP2, thus facilitating the proliferation, invasion, migration, and epithelial–mesenchymal transition of CC cells in vitro, suggesting that *PCA3* may contribute to the progression of CC by acting as a competitive endogenous RNA (ceRNA) against miR-106b.

### 3.5. LINC00261

The long intergenic non-coding RNA 00261 (*LINC00261*) is a lncRNA located on chromosome 20p11.21. Initially, it was found to be differentially expressed in pancreatic and gastric cancers [89,90]. Repression of *LINC00261* results in increased cell proliferation, invasion, migration, and chemoresistance in multiple cancers. This suggests that it plays a tumor suppressor role [91,92,93]. *LINC00261* is downregulated in both CC tissues and cell lines [65]. Furthermore, overexpression of *LINC00261* causes a reduction in cell proliferation, migration, and invasion, and promotes apoptosis in CC JEG-3 and JAR cells. These findings highlight that *LINC00261* may play an important role in the early diagnosis and management of CC.

### 3.6. OGFRP1

Long non-coding RNA OGFRP1 (*OGFRP1*) is a novel lncRNA located on chromosome 22q13.2, and is involved in autophagy regulation in human coronary artery endothelial cells (HCAECs) [94]. It also plays a regulatory role in the proliferative and invasive capacity of various hepatocellular carcinoma (HCC) cell lines, albeit with varying effects in different HCC cell lines [95]. Increased expression of *OGFRP1* has been reported in cervical cancer with subsequent silencing, resulting in the loss of proliferative and invasive capacities of cervical carcinoma cells [96]. These results suggest that in cervical carcinoma, *OGFRP1* might display oncogenic properties. The oncogenic role of *OGFRP1* in CC cells was recently reported by Meng and Xue [66]. Downregulation of *OGFRP1* inhibited cell cycle progression, proliferation, and invasion of JEG-3 and JAR cells and also induced apoptosis through the AKT/mTOR pathway. Even though further research is required to fully understand the role *OGFRP1* plays in tumorigenesis, it appears that *OGFRP1* may be an important therapeutic target in CC.

### 3.7. MIR503HG and LINC00629

*MIR503HG* and *LINC00629* genes, described as long intergenic non-coding RNAs (lincRNAs), are located on Xq26. The same region contains other genes, which are related to the reproductive system, and fetal/placental development [97,98]. Whilst *MIR503HG* expression appears to be restricted to the placenta, *LINC00629* is highly expressed in the placenta as well as reproductive organs [67].

Analysis of RNA-sequencing data sets in colorectal cancer tissues showed an upregulation of *MIR503HG* [99]. Moreover, Chung et al. found that *MIR503HG* is one of the top five non-coding genes, with high levels of expression in anaplastic large-cell lymphoma [100]. In contrast, *MIR503HG* is downregulated in hepatocellular carcinoma [101] and oral squamous cell carcinoma [102]. Fu et al. showed that *MIR503HG* suppresses cell invasion and migration via the miR-103/OLFM4 axis in TNBC [103].

*LINC00629* is downregulated in gastric cancer and RNA *LINC00629* suppresses the progression of this cancer through the upregulation of AQP4, via the competitive binding to miR-196b-5p [104]. Together with *MIR503HG2*, several *LINC00629* isoforms restrain the migration and invasion potential of the JEG-3 tumor cell line. This indicates a potential role for *MIR503HG* and *LINC00629* in tumorigenesis involving the human reproductive system. 

From a treatment perspective, the presence of CpG islands in the promotor regions of both lincRNAs is very interesting since the silencing of promoter regions by DNA methylation is typical of cancer and the subsequent reactivation with DNA demethylating agents could be important in CC treatment. In this regard, Muys et al. demonstrated that overexpression of *MIR503HG2* together with the three new exon *LINC00629* isoforms decreases the migration and invasion potential of the JEG-3 cell line, indicating a possible role for *MIR503HG* and *LINC00629* in CC therapy [67].

## 4. Clinical Applications of lncRNAs in Choriocarcinoma

Given that lncRNAs can be detected in almost all tissues and body fluids, including peripheral blood, and are not easily degraded by RNases, they can be more sensitive and specific than DNA, proteins, and protein-coding RNAs in the diagnosis of tumors [105,106]. Thus, lncRNAs can be potentially utilized as novel non-invasive biomarkers in cancer diagnosis [107]. Even though there has been increasing interest in the study of lncRNAs in gynecological cancers, research is still preliminary. The association of many dysregulated lncRNAs with clinical features leads to the design of novel biomarkers for diagnosis and management of patients with gynecological cancers, ultimately attaining better prognosis [36]. Whether lncRNAs will be also beneficial in the early detection of CC needs to be investigated. Currently, there is no evidence that lncRNAs are used as biomarkers for diagnosis and prognosis of patients with CC. Moreover, given the rarity of the tumor, we hope that these molecules may be used in the future to distinguish gestational CC from non-gestational tumors in an easier and quicker way than the current state-of-the-art methods (biopsy and microsatellite analysis).

## 5. LncRNAs as Therapeutic Targets

LncRNA transcripts have been described as architectural RNAs, molecular scaffolds, and can function as regulatory molecules. They are involved in epigenetic modification, post-transcriptional regulation, mRNA stability, and translation. They may also act as “sponges” for mature miRNAs, whereby they inhibit their activity [108]. Interestingly, most lncRNAs are expressed in a cell type- and tissue-specific manner, and in this respect, lncRNAs lend themselves as potentially important therapeutic targets. Specifically, lncRNA targeted therapy, unlike chemotherapeutic regimens, needs to hinder the signaling pathways in which lncRNA plays an important role in tumor development and progression, whilst aiming to avoid the adverse effects on normal cells. There may also be a potential role of lncRNA as biomarkers for disease management, including non-invasive screening [109].

Recent advances utilize CRISPR technology and oligonucleotide-based therapy to modify gene expression. These approaches may be pivotal for developing novel therapeutic approaches aiming at interfering with oncogenic lncRNAs [110].

The use of RNA interference (RNAi) to modulate gene expression has been successfully used to silence lncRNA in vivo [111]. Advancements in RNAi have led to Food and Drug Administration (FDA) approval of lipid nanoparticles (LNPs) which contain short interfering RNA (siRNA) that is being used to treat hereditary amyloidosis in adults [112]. However, to successfully modulate lncRNA, prior knowledge of lncRNA cellular localization is required. Furthermore, successful implementation requires ensuring that the interfering molecules are stable and, most importantly, their immunogenicity and toxicity is minimal. To ensure stability and avoid enzymatic degradation of RNAi drugs, two modifications have been successfully carried out involving the phosphorothioate backbone [113] and the 2′-*O*-methyl sugar [114].

LncRNAs associated with cancer can also be modulated by using antisense oligonucleotides (ASOs). ASOs are single stranded with a central native or chemically modified DNA stretch, flanked on either side by RNA nucleotides. DNA forms RNA/DNA heteroduplex with target lncRNA, which is cleaved by endogenous RNaseH1 [115]. ASOs have been successfully used to alter mRNA expression as part of the treatment of various diseases, including different types of cancer where lncRNAs are highly expressed [116,117]. Various designs of ASOs are utilized via diverse modes of action, including antagonist to NATs (antagoNAT), locked nucleic acid GapmeRs (LNAGapmeRs), and mixmers.

Advances in genomic interference methods, with superior specificity when compared to RNAi, have been developed recently. CRISPR interference (CRISPRi) using CRISPR-Cas9 and CRISPR-Cas13 led to the successful silencing of transcriptionally active lncRNA-expressing sites. In the CRISPR-Cas9 approach, nucleotides devoid of nucleolytic activity, termed dead-Cas9, are fused to transcriptional repressors. Transcriptional silencing is achieved because, via guide RNAs, this fusion protein is targeted to a specific gene promoter [118]. The development of guide RNAs targeting the promoters of thousands of lncRNAs in the human genome together with CRISPRi enabled the selective inactivation of lncRNA genes in human cancer cell lines. Liu et al. further underscored tissue specificity of lncRNA by identifying approximately 500 lncRNAs in only one cell type [119]. Therefore, it is possible to achieve transcriptional silencing of lncRNAs via CRISPR-based approaches, however, several challenges limit their immediate use for therapeutic targeting [120,121,122,123]. Major limitations include delivery methods (viral and non-viral vectors), with viral vectors limited in the size of the cargo they are able to deliver to the cells of interest [124,125,126,127]. Regardless of the current limitations, the future undoubtedly looks bright for CRISPR-directed lncRNA therapies.

## 6. Future Directions and Conclusions

**Figure 2:**
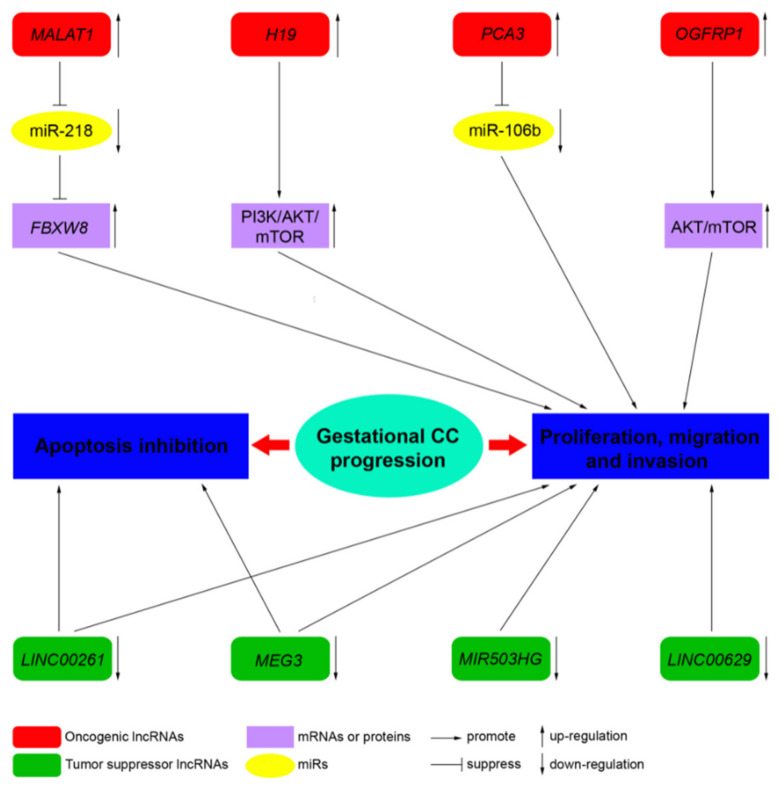
Long non-coding RNAs (lncRNAs) mediate various biological processes to regulate the progression of gestational CC. Certain representative lncRNAs function as oncogenes or tumor suppressors in specific biological processes of gestational CC.

LncRNAs acting as tumor biomarkers have been shown to have an important role in the diagnosis and management of a number of cancers. Studies on the relationship between lncRNAs and gynecological cancer are still at the preliminary stages. Even though different lncRNAs seem to have oncogenic or tumor suppressive roles in CC (Figure 2), the exact underlying molecular mechanism of lncRNAs in CC is largely unknown. A number of lncRNAs could be associated with tumorigeneses, invasion, and stemness, leading to therapy resistance in CC. Currently, research is still in the early stages of characterizing the wide range of roles lncRNAs may play in CC pathogenesis. Therefore, more detailed investigation of lncRNAs in CC is needed. This may revolutionize our understanding of CC and result in advances in management, beyond the state of the art. In this regard, molecular tools including ASOs, siRNAs, and CRISPR technology could be used in the future development of RNA-based therapeutics targeting oncogenic lncRNAs.

## Figures and Tables

**Table 1:** Summary of the features of choriocarcinoma—adapted from Cheung et al. [3].

Types	Gestational Choriocarcinoma	Non-Gestational Choriocarcinoma	
		Germ Cell Tumor	Somatic Carcinoma
Incidence	Ranges from 1 in Europe to −9.2 in Asia/40,000 pregnancies	Rare < 1% of all ovarian tumors—children, young adults but rarely in older adults. Midline tumors mostly in males	Rare ovarian carcinomas in adults
Origin	It may develop as a complication of pregnancy, usually following a complete mole	It arises from primordial germ cells	It arises from differentiation of pluripotent cells into a somatic carcinoma
Site	Primarily uterus and also intraplacental; rarely ovary and extrauterine sites	Gonads, midline: pineal gland, mediastinum, retroperitonum	Lung, gastrointestinal tract, and other organs, including very rare ovarian carcinoma and uterine cases in post-menopause
Histopathology	Mononuclear cytotrophoblast and intermediate trophoblast and multinucleated syncytiotrophoblast cells with marked atypia and mitoses	Mainly in pure form with cyto- and syncytiotrophoblast or with other components of germ cell tumors (mixed germ cell tumor)	Presence hCC-producing multinucleated giant cells; transition with co-existing somatic carcinoma of the particular organ
Cytogenetic features	Deletion of 7p12-7q11.2; amplification of 7q21-q31 and loss of 8p12-21 [3]	Gain of 12p [3]	Unknown
Biochemical features	hCG in serum or urine (>10 × 10^3^ mlU/mL^)^	hCG in serum or urine	hCG in serum or urine—variable
Molecular markers	Upregulation of *TP53*, *CDKN1A*, *RB1*, *EGFR*, *ERBB2*, *c-MYC*, *BCL2*, *NANOG*, *H19* [3,8]; Downregulation of *NECC1*, *TIMP3*, *DOC-2/hDab2*, *RASSF1A*, *CDKN2A*, *CDH1*, *IGF2*, *OCT4*, *SOX2* [3,8]; Mutated genes: *NLRP7*, *ARID1A*, *SMARCD1*, *EP300* [9]	Upregulation of *CGB5*, *CGA*, *NANOG*, *STELLA*, *GDF3* [3]	Upregulation of *NANOG* [3]
Treatment	Chemotherapy	Surgery is indicated. Chemotherapy of different drug regimens is applied	Surgery is indicated. May respond to chemotherapy but it may not be useful
Prognosis	Good	Poor	Poor

**Table 2:** Classification of lncRNAs.

Genomic Location	Description	Example
Intergenic	transcripts originated from intergenic loci; that is, located between two protein-coding genes	*XIST*, *NEAT1*, *PANDAR*, *BGLT3*
Intronic	transcripts originated from introns of protein-coding genes	*NDM29*, *IRAIN*, *EGOT*
Sense	transcripts originated from the sense strand of protein-coding genes, containing exons from protein-coding genes	*SNHG3*, *SRA*, *RUNXOR*
Antisense	transcripts originated from the antisense strand of protein-coding genes	*SNHG6*, *HOXA-AS2*, *ZEB2-AS1*
Enhancer	transcripts, found in both polyadenylated or non-polyadenylated forms, bi-directionally expressed at active enhancer regions of the genome	*DLX6-AS1*, *Alpha-250/Alpha-280*, *LUNAR1*
Promoter	transcripts derived from gene promoter regions in the opposite direction to the paired coding RNA	*DBET*, *pancIl17d*, *HIF2PUT*

**Table 3:** Molecular functions of lncRNAs.

Mechanism Type	Mode of Function	Examples	Reference
Signal	Serves as a molecular signal to reflect development or disease status	*XIST* is typically transcribed by the inactive X chromosome; can be used to indicate X chromosome inactivation	[47,48]
Decoy	Sequestering regulatory factors (transcription factors, chromatin modifiers, miRNAs, etc.) modulate transcription	*PANDAR* inhibits proptosis by directly sequestering NF-YA. *H19* acts as ceRNAs * both for miR-17-5p in thyroid cancer and for miR-152 in breast cancer	[49,50,51]
Guide	Essential for the proper localization of proteins to their site-specific reaction	*MEG3* guides PRC2 and forms a complex with DNA	[52]
Scaffold	Provides platforms to assist in the assembly of regulatory complexes	*HOTAIR* interacts with polycomb repressive complex 2 (PRC2) to recruit EZH2 to promote H3K27 trimethylation or LSD1 to demethylate H3K4me2	[53,54]

* ceRNAs: competing endogenous RNAs regulate other RNA transcripts by competing for shared miRNAs.

**Table 4:** LncRNAs with a role in choriocarcinoma.

LncRNA	Locus	Role	Molecular Functions	Target Pathway	Sources
*MALAT1*	11q13.1	Oncogene	Sponge of miR-218	Unknown	Three CC cell lines, JEG-3, JAR, and BeWo cells, and a normal cell line human trophoblast cells (HT cells) [60]
*H19*	11p15.5	Oncogene	Unknown	PI3K/AKT/mTOR [61]	Placenta, androgenetic moles, and choriocarcinoma [62]; CC cell line JEG-3, including MTX- and 5-FU-resistant variants [61]
*MEG3*	14q32.3	Tumor suppressor	Unknown	Unknown	Placenta; 4 cell-lines associated with pregnancy, including HTR-8/SVneo, JEG-3, WISH, and HUVEC [63]
*PCA3*	9q21-22	Oncogene	Sponge of miR-106b	Unknown	Three CC cell lines, JAR, BeWo, and JEG-3, and the human chorionic trophoblast cell HTR-8 [64]
*LINC00261*	20p11.21	Tumor suppressor	Unknown	Unknown	Sixty CC tissues and 60 adjacent non-cancerous tissues; 3 CC cell lines, namely, BeWo CCL-98, JEG-3, and JAR [65]
*OGFRP1*	22q13.2	Oncogene	Unknown	AKT/mTOR	Two CC cell lines, JEG-3 and JAR [66]
*MIR503HG* and *LINC00629*	Xq26	Tumor suppressor	Unknown	Unknown	RNA samples from a commercial normal human tissue panel and 18 cancer cell lines, JEG-3 cell line [67]

## Data Availability

Not applicable.

## Abbreviations

ASO	antisense oligonucleotide
Cas	CRISPR-associated
CC	choriocarcinoma
ceRNA	competitive endogenous RNA
CRISPR	clustered regularly interspaced short palindromic repeats
CRISPRi	CRISPR interference
FIGO	Fédération Internationale de Gynécologie et d’Obstétrique
GBM	glioblastoma multiforme
HPV	human papillomavirus
LINC00261	long intergenic non-coding RNA 00261
LncRNA	long non-coding RNA
LNAGapmeRs	locked nucleic acid GapmeRs
MALAT1	metastasis-associated lung adenocarcinoma transcript 1
MEG3	maternally expressed gene
NATs	natural antisense transcripts
ncRNA	non-coding RNA
NEAT1/2	non-coding nuclear-enriched abundant transcript ½
NSCLC	non-small-cell lung carcinoma
OGFRP156	opioid growth factor receptor pseudogene 1
PCA3	prostate cancer antigen 3
RNAi	RNA interference
siRNAs	small interfering RNAs
TNBC	triple-negative breast cancer
